# *In Vitro* Studies of the Activity of Dithiocarbamate Organoruthenium Complexes against Clinically Relevant Fungal Pathogens

**DOI:** 10.3390/molecules19045402

**Published:** 2014-04-24

**Authors:** Claudio L. Donnici, Luciano J. Nogueira, Maria Helena Araujo, Sheila Rodrigues Oliveira, Thais F. F. Magalhães, Miriam T. P. Lopes, Ana Cândida Araújo e Silva, Ana Maria da Costa Ferreira, Cleide V. B. Martins, Maria A. de Resende Stoianoff

**Affiliations:** 1 Departamento de Química, Instituto de Ciências Exatas, Universidade Federal de Minas Gerais, Av. Antonio Carlos, 6627, CEP 31270-901, Belo Horizonte, MG, Brazil; 2 Departamento de Microbiologia, Instituto de Ciências Biológicas, Universidade Federal de Minas Gerais, Av. Antonio Carlos, 6627, CEP 31270-901, Belo Horizonte, MG, Brazil; 3 Departamento de Farmacologia, Instituto de Ciências Biológicas, Universidade Federal de Minas Gerais, Av. Antonio Carlos, 6627, CEP 31270-901, Belo Horizonte, MG, Brazil; 4 Instituto de Química da Universidade de São Paulo, Av. Lineu Prestes, 748, CEP 05508-900, São Paulo, SP, Brazil; 5 Gemaq–UNIOESTE, Rua da Faculdade, CEP, 85903-000, Toledo, PR, Brazil

**Keywords:** emerging infectious diseases, invasive fungal infections, dinuclear pentakis-dithiocarbamateruthenium complexes, antifungal susceptibility, cytotoxicity testing, *Candida* spp, *Paracoccidioides brasiliensis*, *Cryptococcus neoformans*, *Sporothrix schenckii*

## Abstract

The *in vitro* antifungal activity of nine dirutheniumpentadithiocarbamate complexes **C1**–**C9** was investigated and assessed for its activity against four different fungal species with clinical interest and related to invasive fungal infections (IFIs), such as *Candida* spp. [*C. albicans* (two clinical isolates), *C. glabrata*, *C. krusei*, *C. parapsolisis*, *C. tropicalis*, *C.dubliniensis* (six clinical isolates)], *Paracoccidioides brasiliensis* (seven clinical isolates), *Cryptococcus neoformans* and *Sporothrix schenckii*. All synthesized complexes **C1**–**C9** and also the free ligands **L1**–**L9** were submitted to *in vitro* tests against those fungi and the results are very promising, since some of the obtained MIC (minimal inhibitory concentration) values were very low (from 10^−6^ mol mL^−1^ to 10^−8^ mol mL^−1^) against all investigated clinically relevant fungal pathogens, except for *C. glabrata,* that the MIC values are close to the ones obtained for fluconazole, the standard antifungal agent tested. Preliminary structure-activity relations (SAR) might be suggested and a strong influence from steric and lipophilic parameters in the antifungal activity can be noticed. Cytotoxicity assays (IC_50_) showed that the complexes are not as toxic (IC_50_ values are much higher—30 to 200 fold—than MIC values). These ruthenium complexes are very promising lead compounds for novel antifungal drug development, especially in IFIs, one of most harmful emerging infection diseases (EIDs).

## 1. Introduction

Emerging infectious diseases (EIDs) are a significant burden on global economies and public health [1]. It is noteworthy that fungi infect billions of people every year, yet their contribution to the global burden of disease is largely unrecognized; in fact, opportunistic invasive fungal infections (IFIs) are a major cause of morbidity and mortality in immunocompromised patients [2,3]. In addition, these severe well-known emerging diseases are difficult to diagnose and subsequent usage of appropriate antifungal therapy is difficult [4]. Besides, this worldwide public health situation has also become alarming [2,3] because of the increase in the frequency of isolation of resistant fungi species even in Brazil [5]. A majority of mycoses-related deaths are associated with *Candida*, *Aspergillus* and *Cryptococcus* spp. infections [6,7]. Among the cited fungi paracoccidioidomycosis (PCM), caused by *Paracoccidioides brasiliensis* is the predominant systemic mycosis in Latin America, causing half of the total deaths from fungal infectious diseases in Brazil. Between 1996 and 2006, most fatal cases occurred among adults between the ages of 30 and 59 years, with approximately 87% of fatalities in men [8]. *P. brasiliensis* is another severe IFI cause, it is sensitive to multiple chemotherapeutic drugs, but very long periods of treatment are expected in these cases, which are monitored by clinical, radiological, and serological follow-up; besides, various interactions of chemotherapeutics against PCM and other drugs are listed in the 2006 PCM guidelines and in several cases the azolic drugs cannot be used [8].

For many years, despite its high toxicity, amphotericin B (AMB) has been the bedrock of systemic antifungal therapy, however fungal resistance has been observed specially in candidiasis cases [9,10,11]. Imidazoles and triazoles (“azoles”) are the largest class of antifungal agents in clinical use [12]. Fluconazole (FLU), the first of them to be used (since 1990), was considered during the 90s “the gold standard” [11,12,13] for the treatment of fungal infections, although due to their indiscriminate use cases of resistance to this drug have been observed [14]. Itraconazole is also very used, especially in the treatment of fungal infections caused by *Aspergillus* and *Sporotrix* that usually are non-suscetible to fluconazole [15], but itraconazole is very hydrophobic, very toxic and drug resistance cases have also been observed to this azole since 1997 [16]. The novel generation of triazoles [17]—voriconazole and posaconazole—approved by FDA in 2002 and 2006, respectively, present a broad action-spectrum against fungal species as *Candida*, *Aspergillus*, *Fusarium*, *Penicillium*, *Scedosporium*, *Acremonium* and *Trichosporon* and it is also active against dermatophytes and dimorphic fungi and *Cryptococcus neoformans* [17,18,19]. Although interactions with other drugs are similar to those ones presented by fluconazole and itraconazole, there are ever fungi resistant to these drugs and also cross-resistance to new generation triazoles [12,14]. Ravuconazole [20] is another of the most successful antifungal class in the clinic and stands out for its unusually long plasma half-life in humans [12], besides it is clearly an “extended-spectrum” triazole with potent *in vitro* activity against these rare and potentially “emerging” opportunistic pathogens [21]. However, it has already been related variable cross-resistance to posaconazole, voriconazole, and even ravuconazole. Cross-resistance between fluconazole and ravuconazole applies most directly to fluconazole-resistant *C. glabrata* and is variable among other species of *Candida* [22]. It is also noteworthy to mention the triazole-resistant fungi description with itraconazole, posaconazole, voriconazole, isavuconazole, or ravuconazole [23]. The last and the newest class of antifungal agents are echinocandins (caspofungin, micafungin and anidulafungin, respectively since 2001, 2005 and 2006) [24,25], that are successors of cilofungin, which was abandoned in the 1980s. In fact, the echinocandins represent the first novel target in 20 years of antifungal drug discovery in terms of clinically useful drugs. The fungal resistance to echinocandins is rare, but the mutation in the target (FKS1) is a common mechanism of the resistance to these drugs [10,26]. This mutation minimizes the drug impact in the fungus cell wall and this has been demonstrated for *C. albicans*, *C. glabrata*, *C. krusei*, *C. tropicalis* and *C. dubliniensis* generating a cross-resistance to all class of echinocandins [27]. The resistance of *C. glabrata* to echinocandins has been associated to FKS2 gene mutation [27] while the resistance to *Cryptococcus neoformans* is related to the different polysaccharide composition of the cell wall in this species [14]. As with all antimicrobial agents, the spectra of emergence of resistance is a real one, and appropriate vigilance in the arms race between fungi and humans means that new targets and new inhibitors will continue to be required for effective antifungal therapy in the future [11,27].

**Figure 1:**
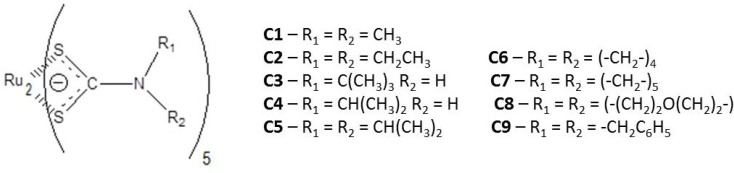
General structure of the studied diruthenium pentakis-dithiocarbamate complexes (C1–C9).

Antifungal agents have also been used in agriculture, among them, the dithiocarbamates are remarkable as worldwide agricultural fungicides, since 1934, when thiram (bis(dimethylthio- carbamoyl)disulfide) was introduced as a seed treatment [28,29,30,31,32]. For this purpose, since 1950s dithiocarbamate complexes mainly with ethylenebisdithiocarbamate (EBDC) as ligand and zinc and manganese as metal center (known as zireb or ziram, and maneb or mancozeb, respectively) have been broadly used [33]. Other dithiocarbamate complexes with different metallic centers and antifungal activity have been investigated [34,35,36,37,38,39,40], but the application of dithocarbamate compounds against human mycoses is relatively rare [38,40]. There is a remarkable study about the antifungal activity of dithiocarbamateorganotin (IV) compounds [41,42] and, besides, there are some studies involving ruthenium coordination compounds activities against filamentous fungi [43,44,45,46,47,48,49]; some of them are related to Ru(II) [36,43,47,49] and others to Ru(III) oxidation states [45,48]. ]. Nevertheless, the first work that describes the investigation of dithiocarbamate ruthenium (III) complexes as antifungal agent [50] has been developed by our own research group, and in this work the susceptibility of seven different species of *Aspergillus* (*A. clavatus, A. flavus, A. fumigatus* (ATCC 16913 and CI, clinical isolated), *A. niger*, *A. nomius*, *A. tamarii* and *A.terreu*s) was tested with promising results considering that *Aspergillus* spp. has also became a recognized pathogen related to IFIs [51,52]. It is remarkable that in some cases [43,50] the ruthenium complexes showed higher activity than the corresponding free ligands. In fact, the study of metal-based drugs represent a therapeutic alternative for novel drug discovery [53,54] and in this way ruthenium complexes provide a rich platform and suitable building blocks for the design of novel bioactive compounds, once the biological activity of the organic ligand is known, due to the specific properties inherent of the transition metal center [55,56]. It is noteworthy that the investigation of novel ruthenium dithiocarbamate complexes as novel and promising drugs is more common for anticancer chemotherapy [55,56,57,58] and also in this area it can be observed that dithiocarbamate chelation with ruthenium can enhance the activity [57] In the present work, nine ruthenium dithiocarbamate compounds (Figure 1, **C1**–**C9**), and their corresponding free ligands, with potential antifungal activity weretested and compared with AMB and fluconazole (FLC) against four different fungal species with clinical interest and related to emerging infectious diseases (EIDs) and invasive fungal infections (IFIs), such as *Candida* spp. (*C. albicans,* two clinical isolates), *C. glabrata*, *C. krusei*, *C. parapsolisis*, *C. tropicalis*, *C. dubliniensis* (six clinical isolates), *Paracoccidioides brasiliensis* (seven clinical isolates), *Cryptococcus neoformans* and *Sporothrix schenckii*.

## 2. Results and Discussion

### 2.1. Chemistry

All dithiocarbamate ligands **L1**–**L9** were synthesized in good yields (63%–93%) as described elsewhere [42,50] through the usual procedure starting from the corresponding substituted amines and carbon disulfide (CS_2_); all ligands were obtained as pure products and have been well-characterized by the usual techniques (IR, ESI-MS, ^1^H and ^13^C-NMR spectra and elemental analysis). The coordination reaction between ruthenium (III) trichloride and the nine recently prepared dithiocarbamate ligands (**L1**–**L9**) (1:3 mol/mol, in ethanol) afforded nine pentakis(dithiocarbamate)diruthenium complexes **C1**–**C9** (**C1**: *N*,*N*-dimethyl**-**; **C2**: *N*,*N*-diethyl-; **C3**: *N*-mono-*tert-*butyl-; **C4**: *N*-mono-(*iso-*propyl)-; **C5**: *N*-di-(*iso-*propyl)-; **C6**: *N*-pirrolidinyl-; **C7**: *N*-piperidinyl-; **C8**: *N*-morpholinyl- and **C9**: *N*,*N*-dibenzyl-dithiocarbamates) in 65%–79% yield. The complete chemical characterization was made by several physicochemical techniques (measurement of magnetic susceptibility (μeff), electron paramagnetic resonance (EPR) spectra, conductivity, cyclic voltammetry) and spectrometric methods (IR, ESI-MS, ^1^H and ^13^C-NMR spectra) [50,59,60,61,62]. All compounds were obtained in high purity level (TLC, HPLC and ESI(+)-MS) and the analysis of the results obtained by ESI-(+)-MS spectrometry suggested the general formula of [Ru_2_(**Ln**)_5_] for all diruthenium pentadithiocarbamate complexes. In fact, the ESI-MS (positive and negative modes) indicated that the compounds are dinuclear neutral species, besides no chloride as counteranion was detected. If the chloride would be present, we should have seen an expected characteristic peak for chloride (^37^Cl/^35^Cl with a 1:3 ratio). The multiple fragmentation pattern observed in the ESI-MS spectra corroborates the obtaining of unsymmetrical dinuclear ruthenium complexes in which the dithiocarbamate moiety plays function as bridging ligand, since the presence of fragments that only can be formed through non-equivalent types of ruthenium-sulfur bonds is noticed, and if the compounds would be symmetrical (as in a mononuclear ruthenium complex or in a kind of oligomeric derivative) the fragmentation pattern would be much less complicated; this discussion will be reported further. There is a recent study [63] about the UV-MALDI mass spectrometry investigation of well-known dithiocarbamate complexes (zinc, iron and manganese derivatives) that corroborates our structural proposal, since in this case symmetrical species (monomeric, dimeric, or tetrameric) were obtained and the corresponding mass spectra are not as complicated, as expected, compared to those in this present study. In addition, the analysis of magnetic susceptibility of the studied compounds showed values consistent with the proposed Ru(II)/Ru(III) system.

**Figure 2:**
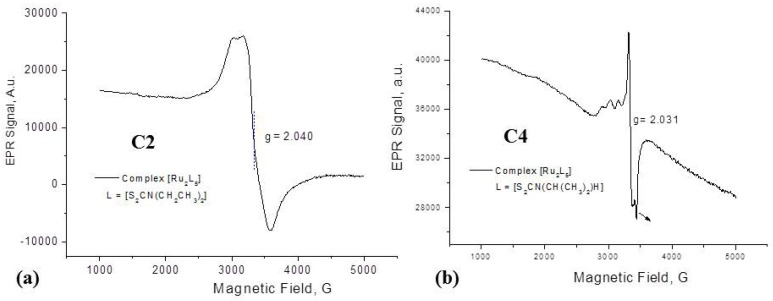
EPR spectra registered in solid state, at 77K, for complexes (**a)** **C2** (α-pentakis(*N*,*N*-diethyldithiocarbamate)diruthenium [Ru_2_(S_2_CN(CH_2_CH_3_)_2_)_5_]) (**b)** **C4** (α-pentakis(*N*-isopropyldithiocarbamate)diruthenium), [Ru_2_{S_2_CN(CH(CH_3_)_2_)(H)}_5_].

The proposal of a dinuclear Ru-Ru system is also corroborated by the infrared bands of ν(Ru-Ru) ≈ 205 cm^−1^) and ν(S-Ru-S) ≈ 280 cm^−1^). The former band associated with ν(Ru-Ru) should be IR-inactive due to the expected high symmetry of the system, nevertheless the observation of this band as cited elsewhere [60] may indicate a distorted octahedral geometry around the diruthenium center. The electrochemical investigation by cyclic voltammetry also indicates that the complexes contain a neutral Ru(II)/Ru(III) system. Analysing the EPR results (Figure 2) for the prepared dithiocarbamate complexes, typical EPR signalw of ruthenium(III) ions in the low spin 4d^5^ configuration can be seen, as shown in Figure 2 (for complexes **C2** (Figure 2a) and **C4** (Figure 2b)); the spike at 3,400 G (*g* ≈ 2.00) in Figure 2b is probably due to an organic decomposition product. It can be suggested that the obtained ruthenium compounds correspond to Ru(II)/Ru(III) mixed-valent states complexes, similar to a species already described in the literature, [Ru_2_(acac)_4_(μ-Q)]^+^, where acac = 2,4-pentanedionate, and Q is a quinonoid group, with two equivalent π-conjugated α-diimine chelate sites, and one *p*-quinone function [64]. Those species can have different oxidation states accessible due to an intramolecular electron transfer, forming [Ru^III^(μ-Q^2^^−^)Ru^II^] or [Ru^II^(μ-Q^•−^)Ru^II^]. In the case of the dithiocarbamate ligands studied, formation of a radical species is also possible, giving rise to [Ru^II^L_2_(μ-L)Ru^III^L_2_] or [Ru^II^L_2_(μ-L^•^)Ru^II^L_2_] complexes.

### 2.2. Antifungal Assays

This study is one of the first approaches to the investigation of the potential usefulness of organoruthenium complexes as antifungal agents that could be used in IFIs. Activities have been determined against a limited number of isolates fungi, but they are representative of important filamentous fungal pathogens in humans.

The antifungal tests were carried out for all ruthenium complexes **C1**–**C9** and the corresponding free dithiocarbamate ligands **L1**–**L9**, against six species (a total of thirteen different strains) of *Candida* spp. (Table 1), the most common fungal pathogen in IFIs [6,7]. In 96% of the cases the ruthenium complexes were more active than the corresponding free ligands (by 3-45 fold). It is important to notice that ruthenium trichloride (control test) did not show any significant antifungal activity for any of the studied microorganisms (>512 μg mL^−1^/196 × 10^−5^ mol L^−1^). The most susceptible species were *C. albicans* ATCC (Table 1) and the clinical isolates 119CL and 01CL. Considering these three species the MIC decreasing activity order is: **C7** > **C6** > **C2** > **C1** > **C5** > **C9** > **C8** > **C3** > **C4**. The most potent complexes **C7** and **C6** showed very low MIC values (0.40 and 0.43 × 10^−5^ mol L^−1^, respectively) that are comparable to the FLU result (0.33 × 10^−5^ mol L^−1^). Then, in a decreasing bioactivity order the complexes: **C2** > **C1** > **C5** > **C9** and **C8** are the most active ones (0.85, 1.0, 2.9, 4.1 and 6.3 × 10^−5 ^mol L^−1^, respectively) and even the last and less potent complexes—**C3** and **C4** ‒ are still quite active (6.8 and 7.33 × 10^−5^ mol L^−1^, respectively). The clinical isolate *C. albicans* 01CL was also very susceptible to the complexes in a similar decreasing (**C7** > **C2** **≥** **C6** > **C1** > **C9** > **C5** > **C4** > **C8** > **C3**) and with close MIC values, although a little higher (1.6–13.6 × 10^−5^ mol L^−1^) when compared to *Candida* ATCC. These data reveal a significant relevancy of these ruthenium complexes as possible novel antifungal agent against IFIs, commonly related to candidiasis, and although AMB could be used, this drug is highly toxic, with low stability and fungal resistance to it has been observed [9,10,11]. It can be noticed that the similar alkyl chain substituted pyrrolidinyl, ethyl, piperidinyl and the methyl derivatives **C7**, **C2**, **C6** and **C1** show the higher activity, while the mono-*N-*alkyl analogues, **C4** (mono-*N*-isopropyl) and **C3** (mono-*N*-isopropyl), and the morpholine derivative (**C8**) are the less active ones.

Among the others studied *Candida* spp. (*C. krusei, C. parapsilosis, C. tropicalis and C. glabrata*) (Table 1), all studied complexes showed also good MIC results: 1.70–7.3, 0.8–4.1, 0.8–7.3 and 4.0–29.3 × 10^−5^ mol L^−1^, respectively and once more the ruthenium complexes were always more active than the corresponding free ligands (by 2–45 fold). Besides, *C. krusei* and *C. parapsilosis* are the most susceptible species and *C. glabrata* is the less susceptible one (Table 1). The most potent complexes against *C. parapsilosis* are **C7**, **C2** and **C6** (MIC: 0.80, 0.85 and 0.86 × 10^−5^ mol L^−1^) and it is observed that these three complexes are almost as potent as FLU (0.33 × 10^−5^ mol.L^−1^), generally the first drug option for drug-resistant fungal disease. Again, similar decreasing activity orders for the studied ruthenium complexes can be pointed out for these three species of *Candida*: *C. krusei*, *C. parapsilosis*, *C. tropicalis*: **C7** > **C2** **≥** **C6** > **C1** **≥** **C9** > **C5** > **C8** > **C4** > **C3**; **C7** **≥** **C2** **≥** **C6** > **C1** > **C5** **≥** **C8** > **C9** > **C3** > **C4**; **C7** **≥** **C2** **≥** **C6** > **C1** > **C3** > **C9** **>** **C5** > **C8** > **C4**, respectively. In the case of *C. glabrata* the activity order for the complexes is completely different: **C1** **≥** **C2** > **C9** > **C5** **≈** **C7** **≈** **C8** **≈** **C6** **≈** **C4** **≈** **C3**.

**Table 1:** *In vitro* antifungal activity of pentakis-dithiocarbamate diruthenium complexes and the corresponding free ligands (MIC, μg mL^−1^/10^−5^mol L^−1^) against different species of *Candida* by microdilution method.

Compounds	*Candida albicans*			*C. krusei*	*C. parapsilosis*	*C. tropicalis*	*C. glabrata*
	ATCC	119CL	01CL				
**L1**	16 (11)	16 (11)	16 (11)	16 (11.2)	128 (89.4)	64 (44.7)	-
**C1**	8.0 (1.0)	16 (2.0)	16 (2.0)	16 (2.0)	16 (2.0)	16 (2.0)	32 (4.0)
**L2**	16 (9.3)	16 (9.3)	16 (9.3)	16 (9.3)	64 (37)	64 (37)	-
**C2**	8.0 (0.85)	16 (1.7)	16 (1.70)	16 (1.70)	8 (0.9)	8 (0.9)	64 (6.8)
**L3**	64 (37)	32 (19)	32 (18.7)	128 (74.7)	32 (18.7)	128 (74.7)	-
**C3**	64 (6.8)	128 (13.6)	128 (13.6)	64 (6.8)	64 (6.8)	32 (3.4)	256 (27.1)
**L4**	32 (21)	16 (10)	16 (10)	16 (10)	32 (21)	64 (42)	-
**C4**	64 (7.3)	64 (7.3)	64 (7.3)	64 (7.3)	64 (7.3)	64 (7.3)	256 (29.3)
**L5**	128 (64)	32 (16)	32 (16)	64 (32)	64 (32)	128 (64)	-
**C5**	32 (3.0)	64 (5.9)	64 (5.9)	32 (3.0)	32 (3.0)	64 (5.9)	256 (23.6)
**L6**	2.0 (1.2)	16 (9.5)	16 (9.45)	8 (4.7)	16 (9.5)	16 (9.5)	-
**C6**	4.0 (0.43)	16 (1.7)	16 (1.71)	16 (1.7)	8.0 (0.86)	8.0 (0.86)	256 (27.4)
**L7**	8.0 (4.4)	16 (8.8)	16 (8.8)	8.0 (4.4)	32 (18)	32 (18)	-
**C7**	4.0 (0.40)	16 (1.6)	16 (1.6)	16 (1.6)	8.0 (0.8)	8.0 (0.8)	256 (25.4)
**L8**	128 (69.1)	128 (69.1)	128 (69.1)	128 (69.1)	128 (69.1)	128 (69.1)	-
**C8**	64 (6.3)	64 (6.3)	128 (12.6)	64 (6.4)	32 (3.2)	64 (6.4)	256 (25.6)
**L9**	8.0 (3.0)	128 (47.9)	64 (24)	8.0 (3.0)	32 (12)	32 (12)	-
**C9**	64 (4.1)	64 (4.1)	64 (4.1)	32 (2.1)	64 (4.1)	64 (4.1)	256 (16.4)
**RuCl_3_**	>512 (196)	>512 (196)	>512 (196)	>512 (196)	>512 (196)	>512 (196)	>512 (196)
**AMB**	0.50 (0.05)	1.00 (0.11)	0.50 (0.05)	0.25 (0.03)	0.50 (0.05)	1.00 (0.11)	0.50 (0.05)
**FLU**	1.00 (0.33)	1.00 (0.33)	32.0 (10.4)	1.00 (0.33)	1.00 (0.33)	32.0 (10.4)	1.00 (0.33)

Considering the antifungal activity tests against *C. dubliniensis* (Table 2), the CD29 and the CD22 clinical isolates were the less susceptible species, but even for CD22 and the complex **C1**, and for **C27** and **C2** the obtained MIC values were comparable, and even lower (8.0 and 6.8 × 10^−5^ mol L^−1^, respectively) than the obtained ones with FLU (10.4 × 10^−5^ mol L^−1^). All complexes and analogous free ligands were also tested against the *C. dubliniensis* CD28 clinical isolate (Table 3). All complexes and all free ligands were active (MIC: 1.7–16.1 10^−5^ mol L^−1^), except **L8**, that showed very low activity (MIC 69.1 × 10^−5^ mol L^−1^). The decreasing activity order of the studied complexes was also similar to the previous ones: **C7** > **C2** **=** **C6** > **C1** > **C5** > **C9** > **C8** > **C4** > **C3**. Although the interesting results obtained, none of them showed lower MIC values than FLU, and as in all cases before, except for *C. glabrata*, the preliminary structure-activity relationship analysis shows that the mono-*N*-alkyl derivatives **C3** and **C4** are the less active compounds and the bis-*N*,*N*-dialkyldithiocarbamate complexes are the most active ones.

In the susceptibility tests of seven clinical isolates of *Paracoccidioides brasiliensis* (Table 4)*,* it can be noticed that the obtained results of the different clinical isolates were quite similar (0.42–12.6 × 10^−5^ mol L^−1^), but the clinical isolate MG05 was the most susceptible species (0.42–1.70 × 10^−5^ mol L^−1^) and that the complexes were also more active than the corresponding free ligands (3–180 fold). 

**Table 2:** *In vitro* susceptibility of species of *Candida dubliniensis* clinical isolates for **L1**, **C1**, **L2** and **C2** complexes by microdilution methods—MIC: μg mL^−1^ (10^−5^mol L^−1^).

Compounds	*Candida dubliniensis*clinical isolates				
	CD22	CD23	CD25	CD27	CD29
**L1**	8.0 (5.6)	4 (2.8)	8 (5.6)	4 (2.8)	8 (5.6)
**C1**	64 (8.0)	8.0 (1.0)	8 (1.0)	8 (1.00)	32 (4.00)
**L2**	8.0 (4.6)	4.0 (2.4)	8.0 (4.8)	8.0 (4.8)	8.0 (4.8)
**C2**	64 (6.8)	64 (6.8)	64 (6.8)	32 (3.4)	64 (6.8)
**AMB**	0.50 (0.05)	1.0 (0.10)	0.50 (0.05)	0.50 (0.05)	1 (0.10)
**RuCl_3_**	>512 (196)	>512 (196)	>512 (196)	>512 (196)	>512 (196)
**FLU**	32.0 (10.2)	1.00 (0.32)	1.00 (0.32)	32.0 (10.2)	1.00 (0.32)

**Table 3:** *In vitro* susceptibility—MIC: μg mL^−1^ (10^−5^mol L^−1^) of *Candida dubliniensis* clinical isolate CD28 for pentakis-dithiocarbamate diruthenium complexes and free ligands by microdilution methods.

	L1	C1	L2	C2	L3	C3	L4	C4	L5	C5
**CD28**	8	16	8	16	16	64	16	64	32	32
	(5.6)	(2.0)	(4.7)	(1.7)	(9.3)	(7.8)	(10)	(7.3)	(16.1)	(3.0)
	**L6**	**C6**	**L7**	**C7**	**L8**	**C8**	**L9**	**C9**	**AMB**	**FLU**
**CD28**	16	16	16	16	128	64	64	64	0.50	1.00
	(9.5)	(1.7)	(8.7)	(1.6)	(69.1)	(6.3)	(24)	(4.1)	(0.11)	(0.33)

**Table 4:** *In vitro* susceptibility of clinical isolates of *Paracoccidioides brasiliensis* for pentakis-dithiocarbamate diruthenium complexes by microdilution methods—MIC: μg mL^−1^ (10^−5^ mol L^−1^).

Compounds	*Paracocidioides brasiliensis* Clinical Isolates						
	MG05	PB01	PB18	PB017	608	B339	MG04
**C1**	16 (2.0)	32 (4.0)	16 (2.0)	32 (4.0)	32 (4.0)	16 (2.0)	16 (2.0)
**C2**	4.0 (0.42)	8.0 (0.85)	16 (1.7)	8 (0.85)	16 (1.7)	4.0 (0.42)	8.0 (0.85)
**C3**	64 (6.8)	64 (6.8)	64 (6.8)	64 (6.8)	64 (6.8)	64 (6.8)	64 (6.8)
**C4**	64 (7.3)	64 (7.3)	64 (7.3)	64 (7.3)	64 (7.3)	64 (7.3)	64 (7.3)
**C5**	64 (5.9)	64 (5.9)	64 (5.9)	64 (5.9)	-	-	- ^a^
**C6**	16 (1.7)	16 (1.7)	16 (1.7)	16 (1.7)	-	-	- ^a^
**C7**	16 (1.6)	16 (1.6)	16 (1.6)	16 (1.6)	-	-	- ^a^
**C8**	128 (12.6)	64 (6.3)	128 (12.6)	128 (12.6)	128 (12.6)	128 (12.6)	128 (12.6)
**C9**	128 (8.18)	64 (4.1)	128 (8.2)	128 (8.2)	128 (8.2)	128 (8.2)	128 (8.2)
**RuCl_3_**	>512 (196)	>512 (196)	>512 (196)	>512 (196)	>512 (196)	>512 (196)	>512 (196)
**AMB**	0.50 (0.05)	1.00 (0.11)	0.50 (0.05)	0.25 (0.03)	0.50 (0.05)	1.00 (0.11)	0.50 (0.05)
**FLU**	1.00 (0.33)	1.00 (0.33)	32.0 (10.4)	1.00 (0.33)	1.00 (0.33)	32.0 (10.4)	1.00 (0.33)

^a^ There was not fungal growth viability.

The complexes **C2**, **C7**, **C6** and **C1** showed the highest activities (respective MIC values: 0.42, 1.6, 1.7 and 2.0 × 10^−5^ mol L^−1^) followed by compounds **C5**, **C3**, **C4**, **C9** and **C8** (5.9, 6.8, 7.3, 8.18 and 12.6 × 10^−5^ mol L^−1^). The four former complexes really showed antifungal activity similar to FLU (0.33–10.4 × 10^−5^ mol L^−1^). These results suggest that the similar derivatives with ethyl (**C2**), tetramethylene and pentamethylene (**C6** and **C7**), and methyl (**C1**) moieties are the most active compounds.

The susceptibility tests of *Cryptococcus neoformans* (Table 5) were also performed and as observed before, the free ligands were less active than the ruthenium complexes (3–11 fold), but when the studied complexes are compared to each other, the results were different than the other ones described above. The *N*,*N*-dibenzyldithiocarbamate diruthenium complex **C9** was the most active agent (MIC: 1.02 × 10^−5^ mol L^−1^) followed by **C5**, **C7**, **C2**, **C6** and **C1** with very similar MIC values (1.48–1.99 × 10^−5^ mol L^−1^) and by **C8**, **C4** and **C3** (3.16, 3.66 and 6.8 × 10^−5^ mol L^−1^). In fact, in this case a preliminary structure-activity relationship shows a clear correlation between the steric hindrance of the *N*,*N*-dialkylsubstituents of the complexes and the antifungal activity: **C9** (*N*,*N*-dibenzyl) > **C5** (*N*,*N*-diisopropyl) ≥ **C7** (piperidinyl) ≥ **C2** (diethyl) ≥ **C6** (pyrrolidinyl) > **C1** (dimethyl). The mono-*N*-alkyl complexes – **C3** (mono-*tert*-butyl) and **C4** (mono-isopropyl) ‒ and the morpholine derivative **C8**, with an oxygen atom in the ring of the substituent, were the less active compounds. It is remarkable that **C9** showed itself almost as potent as FLU (0.65 × 10^−5^ mol L^−1^).

**Table 5:** *In vitro* susceptibility of clinical isolates of *Cryptococcus neoformans* for pentakis-dithiocarbamate diruthenium complexes by broth microdilution methods—MIC: μg mL^−1^ (10^−5^ mol L^−1^).

Compounds	*Cryptococcus neoformans*	Compounds	*Cryptococcus neoformans*
**L1**	16.0 (11.2)	**L6**	16.0 (9.45)
**C1**	16.0 (1.99)	**C6**	16.0 (1.71)
**L2**	16.0 (9.34)	**L7**	16.0 (8.73)
**C2**	16.0 (1.70)	**C7**	16.0 (1.59)
**L3**	16.0 (9.34)	**L8**	64.0 (34.5)
**C3**	64.0 (6.80)	**C8**	32.0 (3.16)
**L4**	16.0 (10.2)	**L9**	128 (47.9)
**C4**	32.0 (3.66)	**C9**	16.0 (1.02)
**L5**	32.0 (16.0)	**RuCl_3_**	>512 (196)
**C5**	16.0 (1.48)	**FLU**	2.0 (0.65)

Only the acyclic derivatives **C1**–**C5** could be tested on *Sporotrix schenckii* (Table 6) since the tests with the other complexes could not be performed due to cell growth problems. The *N*,*N*-dialkyldithiocarbamate ruthenium complexes **C2**–**C1** showed higher activity (MIC: **C2**-0.85 and **C1**-1.0 × 10^−5^ mol L^−1^) followed by the mono-*N*-alkyl analogues **C4** and **C3** (MIC: **C4**-14.8 and **C3**-27.1 × 10^−5^ mol L^−1^); the *N*,*N*-diisopropyl complex **C5** did not show interesting antifungal activity (MIC > 14.8 × 10^−5^ mol L^−1^). **C1** and **C2** indeed showed MIC values that are closely comparable with those obtained with FLU (0.65 × 10^−5^ mol L^−1^).

### 2.3. Cytotoxicity Assay

Results obtained from cytotoxicity assay are summarized in Table 7. The global analysis of the data showed a lower cytotoxicity for complexes **C3**, **C4**, **C5**, **C6**, **C7**, **C8** and **C9**, but the complexes **C1** and **C2** showed a higher toxicity. However, interestingly, even for the most toxic complexes **C1** and **C2** their best *in vitro* antifungal activity was detected at concentrations 90- and 30-fold lower respectively, much smaller values than the IC_50_ obtained on normal mammalian cells.

**Table 6:** *In vitro* susceptibility of clinical isolates of *Sporotrix schenckii* for pentakis-dithiocarbamate diruthenium (**C1**–**C5**) complexes by microdilution methods—MIC: μg mL^−1^ (10^−5^ mol L^−1^).

Compounds	*Cryptococcus neoformans*
**L1**	2.0 (1.4)
**C1**	8.0 (1.0)
**L2**	8.0 (4.7)
**C2**	8.0 (0.85)
**C3**	256 (27.1)
**C4**	125 (14.8)
**C5**	>256 (14.8)
**RuCl_3_**	>512 (196)
**FLU**	2.0 (0.65)

**Table 7:** *In vitro* cytotoxicity of the studied pentakis-dithiocarbamate diruthenium complexes under normal cell line BHK (IC_50_ values in µM).

	C1	C2	C3	C4	C5	C6	C7	C8	C9
BHK-21	89	33	>100	>100	>100	>100	>100	>100	>100

## 3. Experimental

### 3.1. General Information

RuCl_3_·3H_2_O, D_2_O, amphotericin-B (AMB) and fluconazole (FLU) (used as positive control), were purchased from Sigma-Aldrich (St Louis, MO, USA), dimethyl sulfoxide (DMSO; Merck Sharp & Dohme Ltd, Darmstadt, Germany). Other high purity reagents and solvents were used without purification. Dithiocarbamic acid sodium salts (NaS_2_CNR_1_R_2_) were obtained through the classical and usual synthetic route (reaction between carbon disulfide and the corresponding amines) [42,50] and they were used as ligands (**L1**–**L9**) for the synthesis of the corresponding ruthenium complexes **C1**–**C9** that were prepared following a procedure previously reported in the literature [50]. All compounds were fully characterized by the usual spectrometric and physicochemical techniques (IR, ESI-MS, ^1^H and ^13^C-NMR spectra and elemental analysis). ^1^H and ^13^C-NMR spectra were recorded using a Bruker Avance DPX200 MHz spectrometer (Bruker, Karlsruhe, Germany). Chemical shifts (δ in ppm) are referenced to internal solvent resonances and reported relative to SiMe_4_. Infrared spectra were recorded on a Perkin Elmer 283B spectrophotometer (Perkin Elmer, MA, USA), measured in KBr (4,000–400 cm^−1^) and in polyethylene (400–150 cm^−1^) discs. Melting points were determined in the Fisher-Johns equipment and were not corrected. The ESI-MS analyses were conducted on a LCQFleet (Thermo Scientific, San Jose, CA, USA) mass spectrometer bearing an electrospray ionization (ESI) source and operating in the positive ion mode using conditions described elsewhere [65]. EPR spectra were registered in a Bruker EMX instrument (Bruker, Karlsruhe, Germany) [66] operating at X-band (frequency 9.50 GHz, 20.12 mW, and 100 kHz frequency amplitude), using solid samples or frozen solutions, at 77 K, in Wilmad quartz tubes. The magnetic field was calibrated with 2,2-bis(4-tert-octylphenyl)-1-picrylhydrazyl (DPPH) as the external standard (g = 2.0036).

### 3.2. Chemistry: General Procedure for Preparation and Chemical Characterization of Complexes **C1**–**C9** [50]

A reaction mixture consisting of an aqueous solution of RuCl_3_·3H_2_O and the corresponding sodium dithiocarbamate ligand **L1**–**L9** (1:3 stoichiometric molar ratio), was stirred at room temperature for 24 h. The brown solid was collected by filtration, washed with water and dried. The dinuclear ruthenium dithiocarbamate complexes, **C1**–**C9**, obtained as pure compounds (TLC, HPLC and ESI(+)-MS), have been fully characterized (measurement of magnetic susceptibility (μeff), electron paramagnetic resonance (EPR) spectra, conductivity, cyclic voltammetry) [50,59,60,61,62].

α*-Pentakis(N,N-dimethyldithiocarbamate)diruthenium* [Ru_2_(S_2_CN(CH_3_)_2_)_5_] (**C1**): Yield 72%. m.p.: >300 °C; IR(cm^−1^): 1537 m (*N*-CSS), 1012 (C-S), 277 (Ru-S), 209 (Ru-Ru); ^1^H-NMR (200 MHz, DMSO-*d*_6_, δ in ppm): 3.58 (s);^13^C-NMR (50 MHz, DMSO-*d*_6_, δ in ppm): 41.83 (s,CH_3_), 191.75 (s, CS_2_), ESI(+)-MS: *m/z* 803.52 [M^+^] = C_15_H_30_N_5_Ru_2_S_10_Ru_2_ = [Ru_2_(S_2_CN(CH_3_)_2_)_5_]**^+^**.

*α-Pentakis(N,N-diethyldithiocarbamate)diruthenium* [Ru_2_(S_2_CN(CH_2_CH_3_)_2_)_5_] (**C2**): Yield 65%; m.p.: >300 °C; IR (cm^−1^): 1508 m (*N*-CSS), 1009 (C-S), 278 (Ru-S), 209 (Ru-Ru); ^1^H-NMR (200 MHz, DMSO-*d*_6_, δ in ppm): 1.25 (6H), 4.22 (4H); ^13^C-NMR (50 MHz, DMSO-*d*_6_, δ in ppm): 12.90 (s, CH3), 44.33 (s, CH_2_), 195.52 (s, CS_2_); ESI(+)-MS: *m/z* 943.84 [M^+^] = C_25_H_50_N_5_Ru_2_S_10_ = [Ru_2_(S_2_CN(CH_2_CH_3_)_2_)_5_]^+^.

*α-Pentakis(N-tert-butyldithiocarbamate)diruthenium* [Ru_2_{S_2_CN{(C(CH_3_)_3_)(H)}_5_] (**C3**): Yield 71%; m.p.: 240 °C (decomp.); IR (cm^−1^): 1530 (*N*-CSS), 1020 (C-S), 280 (Ru-S), 204 (Ru-Ru); ^1^H-NMR (200 MHz, DMSO-*d*_6_, δ in ppm): 1.49 (s); ^13^C-NMR (50 MHz, DMSO-*d*_6_, δ in ppm): 30.18 (s, CH_3_), 56.81 (s,CHMe_2_), 202.51 (s, CS_2_); ESI(+)-MS: *m/z* 943.85 [M^+^] = C_25_H_50_N_5_Ru_2_S_10_Ru_2_ = [Ru_2_{S_2_CN(C(CH_3_)_3_(H)}_5_]^+^.

*α-Pentakis(N-isopropyldithiocarbamate)diruthenium* [Ru_2_{S_2_CN(CH(CH_3_)_2_)(H)}_5_] (**C4**): Yield 73%; m.p.: >300 °C; IR (cm^−1^): 1513 (C-N), 1030 (C-S), 279 (Ru-S), 203 (Ru-Ru); ^1^H-NMR (200 MHz, DMSO-*d*_6_, δ in ppm): 5.95 (18H), 7.26 (s, 3H); 13C-NMR (50 MHz, DMSO-*d*_6_, δ in ppm): 18.63 (s, CH3), 56.20 (s, CH), 195.01 (s, CS2); ESI(+)-MS: *m/z* 873.83 [M^+^] = C_20_H_40_N_5_Ru_2_S_10_ = [Ru_2_{S_2_CN(CH(CH_3_)_3_(H)}_5_]^+^.

*α-Pentakis(N,N-diisopropyldithiocarbamate)diruthenium* [Ru_2_{S_2_CN(CH(CH_3_)_2_}_5_] (**C5**): Yield 62%; m.p.: >300 °C; IR (cm^−1^): 1483 (N-CSS), 1032 (C-S), 279 (Ru-S), 203 (Ru-Ru); ^1^H-NMR (200 MHz, DMSO-*d*_6_, δ in ppm): 4.84 (36H), 6.85 (m, 6H); ^13^C-NMR (50 MHz, DMSO-*d*_6_, δ in ppm): 13.88 (s, CH3), 56.22 (s, CH), 194.09 (s,CS_2_); ESI(+)-MS: *m/z* 1083.86 [M^+^] = C_35_H_70_N_5_Ru_2_S_10_ = [Ru_2_(S_2_CN(CH(CH_3_)_2_)_5_]^+^.

*α-Pentakis(N,N-dipyrrolidinyldithiocarbamate)diruthenium* [Ru_2_(S_2_C(N(-CH_2_-)_4_)_5_] (**C6**): Yield 78%; m.p.: 210 °C (decomp.); IR (cm^−1^): 1492 (*N*-CSS), 1028 (C-S), 279 (Ru-S), 204 (Ru-Ru); ^1^H-NMR (200 MHz, DMSO-*d*_6_, δ in ppm): 3.3–4.0(NCH_2_), 2.2–1.9(CH_2_); ESI(+)-MS: *m/z* 933.86 [M^+^] = C_25_H_40_N_5_Ru_2_S_10_ = [Ru_2_(S_2_CN(-CH_2_CH_2_-)_2_)_5_]^+^.

*α-Pentakis(N,N-dipiperidinyldithiocarbamate)diruthenium* [Ru_2_(S_2_C(N(-CH_2_-)_5_)_5_] (**C7**): Yield 71%; m.p.: >300 °C; IR (cm^−1^): 1495 (*N*-CSS), 1003 (C-S), 282 (Ru-S), 202 (Ru-Ru); ESI(+)-MS: *m/z* 1003.66 [M^+^] = C_30_H_50_N_5_Ru_2_S_10_ = [Ru_2_(S_2_CN(-CH_2_-)_5_)_5_]^+^.

*α-Pentakis(N,N-morpholinyldithiocarbamate)diruthenium* [Ru_2_{S_2_C(N(-(CH_2_-)_2_O-(CH_2_-)_2_)_5_}_5_] (**C8**): Yield 79%; m.p.: 210 °C (decomp.); IR (cm^−1^): 1484 (N-CSS), 1022 (C-S), 277 (Ru-S), 204 (Ru-Ru); ESI(+)-MS: *m/z* 1005.83 [M^+^] = C_25_H_40_N_5_Ru_2_S_10_ = [Ru_2_(S_2_C(N(-(CH_2_-)_2_O-(CH_2_-)_2_)_5_)_5_]^+^.

*α-Pentakis(N,N-dibenzyldithiocarbamate)diruthenium* [Ru2{S2C(N(CH2C6H5)2}5] (**C9**): Yield 71%; m.p.: 275 °C (decomp.); IR (cm^−1^): 1494 (*N*-CSS), 1010 (C-S), 282 (Ru-S), 202 (Ru-Ru); ESI(+)-MS: *m/z* 1564.16 [M^+^] = C_75_H_70_N_5_Ru_2_S_10_ = [Ru_2_(S_2_C(N(CH_2_C_6_H_5_)_2_)_5_]^+^.

### 3.3. Antifungal Activity

#### 3.3.1. Microorganism Origin

Microorganisms were obtained from the American Type Culture Collection and included *C. albicans* (ATCC 18804) and two clinical isolates:01CL and 119CL), *C. glabrata* (ATCC 2001), *C. krusei* (ATCC 200298)*, C. parapsilosis* (ATCC 20019)*, C. tropicalis* (ATCC 750), *C. dubliniensis* (six clinical isolates: CD22, CD23, CD25, CD27, CD28 and CD29), *Paracoccidioides brasiliensis* (seven clinical isolates: MG05, PB01, PB18, B339, 608, PB1017, MG04), *Cryptococcus neoformans* (ATCC 32608) and *Sporothrix schenckii* (ATCC 10212). The isolates of *Candida* spp. were maintained on Sabouraud dextrose agar (SDA, Difco Laboratories, Detroit, MI, USA) and potato dextrose agar (PDA, Difco Laboratories) to stimulate the conidia production. *S. schenkii* was maintained on BHI medium with glucose 5% (agar and syrup) or RPMI 1640 (Sigma) supplemented with 5% dextrose and *P. brasiliensis* on Fava-netto medium and incubated at 37 °C to obtain the yeast phase and was weekly replicated to maintain the sample viable. 

#### 3.3.2. Susceptibility Testing

Susceptibility tests were performed according to guidelines of CLSI Broth microdilution reference method M27A3 for the yeasts and M38A2 for moulds (CLSI, 20008) on RPMI 1640 with l-glutamine and without bicarbonate (Difco Laboratories) buffered at pH7.0 with 0.165 mol/L [3-(*N*-morpholino)propanesulfonic acid] (Sigma) [67]. The ruthenium complexes **C1**–**C9** and the corresponding free ligands (**L1**–**L9**) were dissolved in DMSO and synthetic RPMI medium. The antifungal tests with the corresponding ligands **L1**–**L9** and even with ruthenium trichloride were also made for comparison. All studied compounds were dissolved in RPMI medium. Serial dilutions were performed using RPMI diluent. One hundred microlitres of each concentration (range 1.0–512 g mL^−1^, or in some cases when specified 0.5–256 g mL^−1^) was distributed in microplates. Stock inoculum suspensions were prepared in sterile saline (0.85%) containing 1% Tween 20 from 7-day-old cultures grown on PDA slants. After heavy particles were allowed to settle, the turbidity of the supernatants was measured spectrophotometrically (Micronal B542, São Paulo, SP, Brazil) at 530 nm, and the transmission was adjusted between 80% and 82%, yielding an initial inoculum of 1–5 × 10^6^ CFU mL L^−1^. Each suspension was diluted 1:50 in medium to obtain twice the desired inoculum (0.5–1 × 10^4^ CFU mL L^−1^). Susceptibility was determined in sterile flat-bottom 96-well microplates. As a control for growth and sterility, RPMI was used without drugs or solvents. Solvent was added to medium as a control for toxicity. All tests were performed in duplicate. The endpoints were determined visually by comparison with that drug free growth control experiment. Minimum inhibitory concentrations (MICs) were defined as the lowest drug concentrations that provided for which optically clear, except for Fluconazole (FLU) (endpoint allowing 50% reduction in growth) and they were expressed in μg L^−1^. Amphotericin B (AMB) and Fluconazole (FLU) were included as positive antifungal controls. Stock solution was prepared by dissolving the drugs in RPMI at a concentration of 1.5 mg L^−1^. For antifungal testing, concentrations range 0.03–16 μg L^−1^ to AMB and 0.25–128 μg L^−1^ to FLU were used. The results were read after 48 h incubation at 35 °C.

#### 3.3.3. Cytotoxicity Assay

Cell Cultures: 

BHK-21 cells (Baby Hamster Kidney epithelium) were grown in T-25 flasks in 5.0 mL of RPMI medium (Sigma-Aldrich) containing 10% fetal calf serum (Cultilab, São Paulo, SP, Brazil) and supplemented with antibiotics ampicillin (100 U mL^−1^) and streptomycin (100 mg mL^−1^). The cells were maintained in the logarithmic phase in 5% CO_2_ atmosphere at 37 °C and subcultured by harvesting with 0.05% trypsin-ethylenediaminetetraacetic acid (EDTA) solution.

Cytotoxicity Assay Experiments: 

The cytotoxic activity of the compounds towards normal cell line was evaluated through the 3-(4,5-dimethylthiazol-2-yl)-2,5-diphenyltetrazolium bromide (MTT) assayv[68]. Briefly, 1.0 × 10^3^ cells in 100 μL of culture medium were seeded in 96-well microplates and cultivated as described above. After 48 h, the medium was removed and replaced with a fresh one containing different concentrations (10^−7^ to 10^−4^mol L^−1^) of studied compounds prepared in sixplicate. After 120 h, each well was treated with 10 μL of 5 μg mL^−1^ MTT aqueous solution and after 4 h of incubation, the medium was aspirated and 100 μL of DMSO was added. The cytotoxic effect induced by compounds was detected by measuring the absorbance at 570 nm using a Stat Fax-2100 (Awareness Technology Inc., Palm City, FL, USA) microplate reader. Mean absorbance for each compound concentration was expressed as a percentage relative to the control group (untreated cells), and plotted *vs.* concentration. IC_50_ values represent the drug concentrations that reduced the mean absorbance at 570 nm to 50% of those in the untreated control wells (or determination of inhibitory concentration to 50% cell viability).

## 4. Conclusions

In this work, we synthesized and characterized a set of nine dithiocarbamate ligands and the nine corresponding dirutheniumpentakis-dithiocarbamate complexes in good yields. All synthesized complexes (**C1**–**C9**) (**C1**: *N*,*N*-dimethyl-; **C2**: *N*,*N*-diethyl-; **C3**: *N*-mono-*terc-*butyl-; **C4**: *N*-mono-(*iso-*propyl)-; **C5**: *N*-di-(*iso-*propyl)-; **C6**: *N*-pyrrolidinyl-; **C7**: *N*-piperidinyl; **C8**: *N*-morpholinyl-and **C9**: *N*,*N*-dibenzyl-dithiocarbamates) were submitted to *in vitro* tests against some fungi with clinical interest. Except for *Candida glabrata*, the less susceptible fungal species, all other species related to IFIs showed high susceptibility to almost all studied complexes and with good antifungal activity results (MIC values in the order of 10^−5^ mol L^−1^ to 10^−8^ mol mL^−1^). In some cases, the obtained MIC values were close or even lower than the obtained MIC value for the classic clinically used antifungal agent, Fluconazole (FLU). For instance, considering candidiasis and paracoccidioidomycosis that are usually related to IFIs in Latin America, the diethyl- (**C2**), the pyrrolidinyl- (**C6**) and the piperidinyl-(**C7**) diruthenium complexes show remarkably antifungal activities, while for cryptococcosis cases the *N*,*N*-dibenzyl complex (**C9**) might be indicated and for sporotrichosis, the simpler *N*,*N*-dimethyl- (**C1**) or *N*,*N*-diethyl-(**C2**) analogous complexes can be used. The results showed that the complexes are more active than the corresponding free ligands and these data can be explained considering the higher lipophilicity of the complexes [53]. Preliminary structure-activity relations (SAR) suggest a strong influence from steric and lipophilic parameters in the antifungal activity of the studied ruthenium complexes and these data are corroborated by a quite recent QSAR study [69] in which it was reported the dependence of the antifungal activity from several known antifungal agents on the lipophilic and steric effects. Cytotoxic assays (IC_50_) showed that the complexes are not so toxic (IC*_50_* values are much higher than MIC values). Finally, it can be concluded that ruthenium dinuclear pentakis-dithiocarbamate complexes can be considered as potential novel antifungal agents with high potency and low citoxicity for the development of new drugs to attack the terrible worldwide health problem of invasive fungal infections (IFIs) and emerging infectious diseases (EIDs). 
